# Effects of exercise on pain of musculoskeletal disorders: a systematic review

**DOI:** 10.1590/1413-78522014220601004

**Published:** 2014

**Authors:** Elisângela Valevein Rodrigues, Anna Raquel Silveira Gomes, Aldre Izabel Pchevozniki Tanhoffer, Neiva Leite

**Affiliations:** 1. Universidade Federal do Paraná, Curitiba, PR, Brazil, Physical Education, Universidade Federal do Paraná (UFPR), Curitiba, PR, Brazil; 2. Instituto Federal do Paraná, Curitiba, PR, Brasil, Instituto Federal do Paraná (IFPR), Curitiba, PR, Brasil

**Keywords:** Exercise, Pain, Musculoskeletal diseases, Workplace

## Abstract

Work related musculoskeletal disorders are a major concern for public health and pain is the most important symptom. The aim of this study was to verify the effectiveness of workplace exercises to control musculoskeletal pain and its frequency, intensity, duration and type of exercises used. The search was conducted systematically in Medline, Pubmed, Embase, Bireme, Web of Knowledge and Pedro databases. The keywords "workplace", "exercise" and "musculoskeletal disorders" were used combined. Randomized control trials which performed worksite exercises were selected and the studies were assessed by their methodological soundness. Ten articles were selected which investigated the resistance training, cardio respiratory exercises, Pilates, stretching, postural orientation and exercises for relaxation. Workplace resistance training performed at 70-85% RM, three times a week for 20 minutes promotes reduction of the pain in shoulders, wrists, cervical, dorsal and lumbar spine. However, there is no consensus regarding the total duration of the intervention for the decrease of musculoskeletal pain in these regions. **Level of Evidence I, Therapeutic Studies Investigating the Results of Treatment, Systematic Review of RCTs (Randomized and Controlled Clinical Studies).**

## INTRODUCTION

The work-related musculoskeletal disorders (WRMD) are a worldwide public health problem and often can lead to temporary or permanent disability at work.^1^ They are considered multifactorial, complex and of insidious nature. It is a clinical syndrome characterized by pain in the lower back, neck, shoulder girdle and upper limbs, accompanied or not by lesions in tendons, muscles and peripheral nerves.^2^^,^ 3 Musculoskeletal disorders in the lower back and upper limbs have reached epidemiological proportions, causing high costs for the global economy, due to the costs dispensed with health care, worker's compensation insurance and sick leave, and it is considered one of the three largest causes of absence from work.^4^^,^ 5 The decrease in productivity due to absence from work, chronic diseases and health expenditures lead to estimated annual spending of 2.1 billion Euros in the Netherlands and about 45 to 54 million Dollars in the United States.^4^

The etiology of WRMD is considered complex due to the presence of several factors such as individual factors related to gender and comorbidities; physical factors; organizational factors; overwork and also ergonomic factors; psychological and sociais factors.^1^^,^ 6 However, it is known that the overuse of certain muscle groups, performing repetitive movements with or without required located effort, postures during work, short rest interval and stress imposed by the work organization predisposes musculoskeletal dysfunctions.^7^^,^ 8

Pain is one of the major symptoms related to musculoskeletal disorders with difficult clinical management because it is felt individually.^4^ Among the strategies that have been used, resistance, stretching and cardiovascular fitness exercises have been conducted in occupational settings in order to reduce the pain and improve health, work ability and quality of life of the workers. However, the effects of exercise performed in the occupational environment regarding pain improvement are inconclusive.^9^

The aim of this study was to verify the effectiveness of exercise programs in the occupational environment in controlling musculoskeletal pain. Moreover, it also aimed to verify the influence of the type of exercise, intensity, frequency and duration of the training for reducing musculoskeletal pain.

## METHODS

Studies that investigated participants with musculoskeletal pain were included; with intervention containing exercises performed individually or in a group with a structured program; exercises performed in the workplace; articles in English and Portuguese; documents and full text articles freely available.

Exclusion criteria were studies that evaluated the effect of changes in risk factors, occupation and other factors that triggered musculoskeletal pain; intervention programs without monitoring by experienced profissional; intervention associated with supplementation or medication program; review articles, letters to the editor, comments, study protocols, case studies, theses and dissertations; studies performed over 10 years ago.

### Search strategy and selection of studies 

The search for articles was conducted systematically in the following electronic databases: Medline, Pubmed, Embase, BIREME, Web of Knowledge and Physiotherapy Evidence Database (PEDro). The descriptors "workplace", "exercise" and "musculoskeletal disorders", which are present as Health Science Descriptors (DeCS), were combined together with the Boolean operator "AND".

Two researchers (E.V.R and A.I.P.T) independently and in duplicate, first assessed the titles and then abstracts. All titles and abstracts which fulfilled the inclusion and exclusion criteria were selected for full reading. At each stage the differences between raters were treated by consensus.

### Extraction of Data 

The data extraction was performed by the same two reviewers independently and differences were resolved by consensus. We used standardized forms that included the first author, year of publication, research subjects, groups and outcomes. The variables of interest extracted were methods used in studies for the evaluation of the outcomes.

### Assessment of risk of bias and Jadad classification 

The studies considered for the analysis were reviewed by two reviewers independently.^10^ The assessment of bias risk of the studies was performed using the Jadad score ranging from 0 to 5 and the studies were classified in high quality (score 3-5) and low quality (score 1-2). This index finds a specific value for each of the five following factors: the radomization of the study, if the study was double blinded, if the losses were described, if the randomization and masks were performed properly.^11^ Researches that used, in the randomization, a method of generating random sequence were considered appropriate and those without clear description of randomization or sequence generation methods using date of birth, date of admission, hospital record number or alternation between groups were considered inadequate.^10^^-^ 12

## RESULTS

The association of the three descriptors *("workplace", "exercise" and "musculoskeletal disorders")* generated 349 results, whose 59 were excluded. Fifty-two were repeated, four for being in German language and three for having been published over 10 years before the beginning of the research.

**Figure 1:**
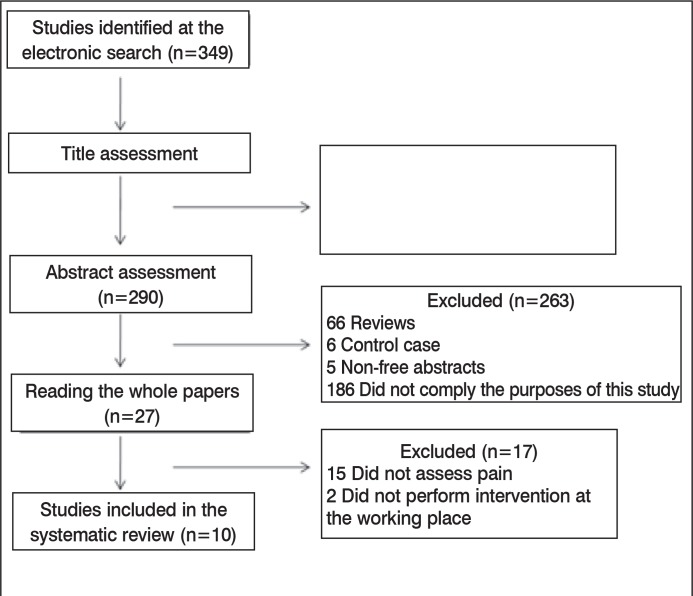
Selection process of studies included in the review

Of the 290 abstracts assessed, 66 were systematic reviews; five abstracts freely available; six control cases; and 186 articles have not studied the effect of exercise on musculoskeletal pain, totaling 263 articles eliminated. Thus, 27 were eligible for the study and were read in full. Of these, fifteen studies were eliminated because they have not examined the effect of exercise on pain; and other two studies were excluded because the exercises were performed outside the workplace. Thus, ten studies were considered in this systematic review. The flowchart of the study is shown in Figure 1.

The results of this review are described in chronological order in Table 1 including the items: author, year of publication, the research subjects (number, age, gender) design, interventions and variables.

**Table 1:** Characteristics of the analyzed studies.

Authors, year	Type of study	Sample (n)/Gender/ Occupational function	Intervention group	Control group	Variables studied
Sjogren *et al*., 2005^13^	RCT* - cross-over*	n=53 43 women 10 men - Office workers	EG1, n=36 EG2, n=17	CG2, n=17 CG1, n=36	Pain: Borg Scale (CR10); Sub-maximum test of 5 RM on resistance equipment (HUR Ltd, Finland).
Sjogren *et al*., 2006^14^	RCT* - cross-over*	n=36 29 women and 7 men Office workers	EG1, n=21 EG2, n=15	CG2, n=15 CG1, n=21	Pain: Borg Scale (CR10); Sub-maximum test of 5 RM on resistance equipment (HUR Ltd, Finland).
Andersen *et al*., 2008^15^	RCT	n=42 women Office workers	STG, n=18 VTG, n=16	Couns G, n=8	EVA – Acute and Chronic pain; CVIM; Åstrand Method – aerobic aptitude (VO_2max_).
Andersen *et al*., 2008b^16^	RCT	n= 549 Women e men Office workers	RG, n=180 VTG, n=187	Couns G, n= 182	VAS - pain MIVC.
Pedersen *et al*., 2009^17^	RCT	n= 549 Women and men Office workers	RG, n=180 VTG, n=187	Couns G, n=182	IPAQ; Musculoskeletal pain questionnaire; General Health and Productivity; MIVC; Oxygen uptake
Andersen *et al*., 2010^18^	RCT	n= 549 Women and men Office workers	RG=180 VTG=187	Couns G, n=182	Pain: 0-9 scale, Pain location - Nordic Questionnaire; MIVC
Jay *et al*., 2011^19^	RCT	n= 40 Women: 34 and Men: 6 Pharmaceutical industry lab workers	EG=20	CG=20	VAS: Pain; MIVC; Åstrand Protocol: VO_2Max_; BP
Zebis *et al*., 2011^20^	RCT	n= 448 Women e men Lab technician	EG=211	Couns G=237	Strength: UL with weight – RM; Nordic Questionnaire – pain regions; Pain scale (0-9)
Macedo *et al*., 2011^21^	Non-randomized CT	n= 50 Office workers	EG=29	CG=21	VAS, pain regions
Marangoni, 2010^22^	RCT	n= 68 Women and men Office workers	Exercise Group 1 (EG 1) = 22 (online) Exercise Group 2 (EG 2) = 23 (printed)	CG=23	VAS; Pain location: Human body drawing; Daily index of pain; Pain points index

RCT: Randomized and Controlled Clinical Trial; CT: Clinical Trial; EG: Exercise Group; CG: Control Group; STG: Specific Training Group; VTG: Varied Training Group; COUNS G: Counseling Group; RG: Resistance Group; RM - Repetition Maximum; VAS - Visual Analogue Scale; MIVC: Maximum Isometric Voluntary Contraction; IPAQ: International Physical Activity Questionnaire; BP; Blood pressure; UL: Upper limbs.

### Modalities and exercise protocols used 

The modalities of exercises found in the articles were: strength training,^13^^-^ 20 cardiorespiratory fitness,^15^^,^ 16 ^,^ 18 Pilates,^21^ stretching exercises,^21^^,^ 22 and relaxing exercises.^21^

Strength training was performed with dumbbells,^15^^-^ 18 ^,^ 20  resistance apparatus,^13^^,^ 14 and ketlebell.^19^ The load varied from 30% in one maximum repetition (1 repetition maximum (RM)),^13^^,^ 14 70-80% of 1RM;^15^^-^ 19 and 70-85% of 1RM.^20^

The cardiorespiratory fitness was performed with an ergometric bike^15^ and plyometric rowing equipment and kaiake.^16^^,^ 18 Pilates and relaxing exercises^21^ were not specified.

Among the 10 studies analyzed, nine were randomized control trials (RCT),^13^^,^ 20 ^,^ 22 two using cross-over design^13^^,^ 14 and one was not randomized.^21^ Four studies had control groups that received health and ergonomics guidelines^15^^-^ 18 and in six studies non intervention was applied in the control group.^13^^,^ 14 ^,^ 19 ^-^ 22

The duration of training programs ranged from 15 days to 12 months. Regarding the weekly frequency, interventions were evaluated five times week,^13^^,^ 14 in 15 working days^22^ and the frequency of three week sessions.^15^^-^ 21 The duration of the interventions was twenty minutes,^13^^-^ 20 fifteen minutos^21^ and one study conducted intermittent intervention every six minutes during working time.^22^ The week days that workouts were applied, as well as the time of day in which the subjects performed the intervention were not specified.

### Assessment of pain and other variables

Pain was assessed by using the visual analogue scale (VAS), using a scale from zero to ten^15^^,^ 16 ^,^ 19 ^,^ 21 ^,^ 22 and zero to nine,^18^^,^ 20 Borg pain scale (CR10)^13.14^ and structured questionnaire of pain frequency.^17^

Two studies used the design of a human body to the location of pain areas with the quantification of the intensity of pain.^21^^,^ 22 Two studies^18^^,^ 20 used the Nordic questionnaire for the identification of pain in the neck, shoulders, back, elbow, wrists/hands, lower back, hips/ thighs, knees and ankles/feet. The other assessed pain in the cervical spine, shoulder and lumbar spine;^17^^,^ 19 cervical spine and shoulder;^13^^,^ 16 trapezius region;^15^ and lumbar spine.^14^

### Risk of bias and Jadad score 

Of the studies included in the systematic review, 90% underwent randomization; 60% described the method used in randomization; no study was double-blind; 90% reported losses in monitoring and exclusions. (Table 2)

**Table 2:** Risk of bias of included studies and Jadad scores.

Study and reference	Randomization	Double blind	Description of losses	Appropriate randomization	Appropriate double blind	Jadad classification
Sjögren et al.^13^	Yes	No	Yes	No	No	Low quality
Sjögren et al.^14^	Yes	No	Yes	No	No	Low quality
Andersen et al.^15^	Yes	No	Yes	Yes	No	High quality
Andersen et al.^16^	Yes	No	Yes	Yes	No	High quality
Pedersen et al.^17^	Yes	No	Yes	Yes	No	High quality
Andersen et al.^18^	Yes	No	Yes	Yes	No	High quality
Jay et al.^19^	Yes	No	Yes	Yes	No	High quality
Zebis et al.^20^	Yes	No	Yes	Yes	No	High quality
Macedo et al.^21^	No	No	Yes	No	No	Low quality
Marangoni^22^	Yes	No	No	No	No	Low quality

It was found that of the ten papers, regardless of the classification received in the Jadad scale, all showed control groups and similarity of the pre-intervention groups. Only two had sample calculation, three had follow-ups and eight studies showed regular and supervised program.

## DISCUSSION

The primary outcome found in this systematic review on the effects of regular and targeted exercise conducted in the workplace, was the improvement of pain in shoulders, wrists and spine. However, there was no consensus regarding the parameters of prescribing exercises for the improvement of musculoskeletal disorders in these regions.

Therefore, this review also examined other issues that concern the influence of the type of exercise, intensity, frequency and duration of the training in pain outcome. Of the ten studies analyzed in this review, all showed significant improvements in reducing pain in the trapezius muscle in shoulders, wrists, cervical, dorsal and lumbar spine.

Muscle strength had mixed results, probably due to differences in the intensity of the exercises used for training of muscle strength, around 70-85% of RM^15^^-^ 20 and 30% of 1RM in two studies of Sjögren *et al*.^13^^,^ 14 However, such protocols meet the recommendations of the American College of Sports Medicine for resistance exercises.^23^ Despite the variations found in the protocols used in each study training, pain improvement was observed in all training intensities investigated.

Non-specific exercises for the pain location as stationary bike,^15^ plyometric paddling devices and kaiake,^16^^,^ 18 Pilates and relaxation exercises,^21^ as well as stretching exercises^21^^,^ 22 also promoted decrease of pain.

In this review it was found that office workers were the most studied population.^13^^-^ 18 ^,^ 21 ^,^ 22 These exert their occupational activities mainly seated and using the computer, which generates pain mainly in shoulders and the cervical region.^24^ The physical exercise applied to this population promoted lifestyle changes, which may have contributed to the reduction of musculoskeletal pain. Bernards *et al*.^4^ found that the change of lifestyle both at work and in free time as behavioral changes, adjustments in the workplace, work breaks and exercise in their free time promoted improvement of pain in office workers.

The methods of analysis of the symptom of pain varied among studies, however, it can be seen that five studies that analyzed the pain intensity, VAS was used with a zero to ten score.^15^^,^ 16 ^,^ 19 ^,^ 21 ^,^ 22 All studies justifying the use of VAS as a tool for secure and reliable assessment to be validated internationally, besides being easy to use and to understand by participants.

Regarding the methodological quality of studies, every high quality study^15^^-^ 20 performed strength training as an intervention for reducing pain. In three of them, the duration of the study was 12 months,^16^^-^ 18 in the others, duration varied between eight,^19^ ten,^15^ and 20 weeks.^20^ All of them underwent training three times a week for 20 minutes and the intensity ranged 70-85% of RM. Low quality methodological studies used muscle training^13^^,^ 14 with 30% of RM, with daily frequency, for 20 minutes for 15 weeks; stretching exercises,^22^ intermittently 10 to 15 seconds during 15 days; and 15 minutes of stretching, relaxation and Pilates^21^ three times a week for eight months.

Thus, the findings showed that strength exercises performed with high intensity (70-85% of RM), three times a week for 20 minutes reduce pain in workers. However, further studies are essential to investigate which intervention duration (weeks or months) is necessary to promote the reduction of pain, since time varied between studies. Moreover, it is necessary to undertake studies focused to the same training protocols for the same painful region, because regions varied in high quality studies (neck, shoulder, thoracic spine, lumbar spine, trapeze, wrists, thighs, ankles and feet).

To practice stretching exercises, studies should conduct more structured and rigid duration and intensity protocols and intervention period to verify their effects in decreasing musculoskeletal pain.

Furthermore, the sample size calculation is important for proper sizing of the sample, the inference of the data obtained clinically, besides ethical and economic issues, which contributes to improving the quality of the study.

Another factor that must be taken into account is the follow-up, to investigate the effectiveness and duration of benefits acquired with the exercises. This criterion has clinical as well as economic relevance, due to costs dispensed with health care, compensation, and sick leave.

## FINAL CONSIDERATIONS

Strength exercises with intensity of 70-85% of RM performed in the workplace, three times a week for 20 minutes are able to reduce musculoskeletal pain in shoulders, wrists, cervical, thoracic and lumbar spine. However, there was no consensus regarding the total duration of exercise program to improve musculoskeletal pain in different body regions studied.
